# Pollutant Photodegradation Affected by Evaporative Water Concentration in a Climate Change Scenario

**DOI:** 10.3390/molecules29112655

**Published:** 2024-06-04

**Authors:** Arianna Rosso, Davide Vione

**Affiliations:** Dipartimento di Chimica, Università di Torino, Via Pietro Giuria 5, 10125 Torino, Italy; arianna.rosso336@edu.unito.it

**Keywords:** pollutants, photochemical reactions, natural attenuation processes, surface waters, global warming, water scarcity, precipitation decrease, water evaporation

## Abstract

Evaporative water concentration takes place in arid or semi-arid environments when stationary water bodies, such as lakes or ponds, prevalently lose water by evaporation, which prevails over outflow or seepage into aquifers. Absence or near-absence of precipitation and elevated temperatures are important prerequisites for the process, which has the potential to deeply affect the photochemical attenuation of pollutants, including contaminants of emerging concern (CECs). Here we show that water evaporation would enhance the phototransformation of many CECs, especially those undergoing degradation mainly through direct photolysis and triplet-sensitized reactions. In contrast, processes induced by hydroxyl and carbonate radicals would be inhibited. Our model results suggest that the photochemical impact of water evaporation might increase in the future in several regions of the world, with no continent likely being unaffected, due to the effects of local precipitation decrease combined with an increase in temperature that facilitates evaporation.

## 1. Introduction

Contaminants of emerging concern (CECs) are harmful chemicals that are often emitted by wastewater treatment plants (WWTPs) [1]. WWTPs have difficulties in removing those CECs that are biorecalcitrant and water-soluble, which allows them to minimize both biodegradation and partitioning on biosolids in the activated sludge biological step. Therefore, CECs mainly remain in the aqueous phase from where they can easily reach surface waters [1,2]. There, CECs and their transformation products are harmful to aquatic life forms and potentially also to human health through use of water for irrigation, recreational activities, and as a source of drinking water [3,4,5]. CECs include a wide list of compounds, such as pharmaceuticals, personal care products, pesticides, and industry-relevant intermediates [1].

The biorecalcitrance of many CECs is also a problem in the natural environment. Although biodegradation in surface waters might be different from WWTP processes [6,7], compounds surviving biological WWTP treatment often undergo poor biodegradation in aquatic ecosystems as well. Photodegradation can thus become a competitive pathway for the elimination of these compounds from surface waters [8]. Photochemical CEC attenuation involves direct photolysis and indirect photodegradation. In direct photolysis, the CEC absorbs sunlight and is promoted to an electronically excited state that undergoes degradation by bond breaking, photoionization, reaction with the solvent, or with reactive solutes. In the case of indirect photochemistry, sunlight is absorbed by chromophores called photosensitizers, most notably nitrate, nitrite, and chromophoric dissolved organic matter (CDOM) [8,9,10]. Radiation absorption by photosensitizers yields photochemically produced reactive intermediates (PPRIs), which include hydroxyl (^•^OH) and carbonate (CO_3_^•−^) radicals, singlet oxygen (^1^O_2_), and the excited triplet states of CDOM (^3^CDOM*). PPRIs reach steady-state concentrations of about 10^−18^–10^−14^ M, which result from the budget between photogeneration and scavenging/quenching [11]. In particular, ^•^OH is photochemically generated by CDOM, NO_3_^−^, and NO_2_^−^ and is scavenged by DOM (the dissolved organic matter, not necessarily chromophoric, quantified as dissolved organic carbon (DOC)), by HCO_3_^−^, CO_3_^2−^, and, to a lesser extent, NO_2_^−^. However, in brackish waters and seawater, most of the ^•^OH scavenging is carried out by Br^−^ [11,12]. The oxidation of HCO_3_^−^/CO_3_^2−^ by ^•^OH and the oxidation of CO_3_^2−^ by ^3^CDOM* yield CO_3_^•−^, which is mainly scavenged by DOM. CDOM is the only source of ^3^CDOM*, which is mostly quenched by O_2_ to give ^1^O_2_ with ~50% yield, while ^1^O_2_ is mainly quenched by collision with water [13,14,15]. DOM is usually not a significant scavenger of either ^3^CDOM* or ^1^O_2_ for DOC < 20 mg_C_ L^−1^ [11].

Photodegradation kinetics are highly affected by water chemistry and depth, which are in turn altered by climate change [11]. In particular, a strong link is expected between water scarcity and photodegradation processes in both rivers and lakes [11,16]. Enhanced CEC photodegradation would partially offset the fact that, if water is scarce, wastewater undergoes little dilution, with increased pollution as a consequence [17].

Evaporative water concentration occurs in stationary water bodies, such as lakes and ponds, when evaporation prevails over outflow or seepage and when the water level is not regenerated by precipitation. Evaporation often occurs in arid and semi-arid environments and sometimes proceeds up to complete desiccation [18,19]. In the process, photochemical reactions are deeply affected, and different photoinduced pathways are modified to different extents [11,20]. This work has the goal of understanding how different CECs would behave under evaporative concentration conditions. We carry out this task by photochemical modeling and also try to understand if, by the end of the century, there is potential for the phenomenon to increase in importance on a worldwide scale.

## 2. Results and Discussion

### 2.1. Water Loss by Evaporation in Model Lakes

When water evaporates from a lake, there is loss of volume and of volatile solutes, but non-volatile compounds, such as soluble ions, remain in the residual water and become concentrated as a consequence. The extent to which they are concentrated and the photochemical implications of the process depend on the way the evaporation of water affects lake depth and volume, which in turn depend on the lake geometry [11].

**Figure 1:**
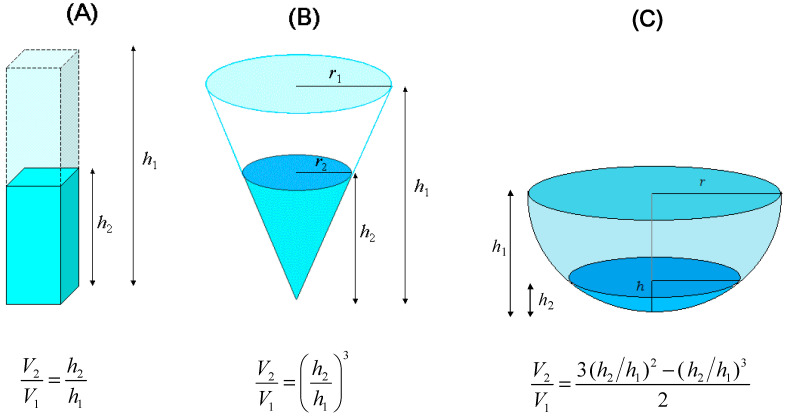
Geometry of water loss, depending on model lake shape. (**A**) Swimming pool-like system. (**B**) Conical system. (**C**) Hemispherical system. For simplicity, here we assumed a scenario that can be assimilated to case (**B**). V = volume.

The simplest geometrical scenario is relevant to a system that resembles a swimming pool with vertical slopes (Figure 1A). In this case, the surface area *S* is not modified by evaporation, and the volume is proportional to the height of the parallelepiped (*V*_1_ = *S h*_1_, *V*_2_ = *S h*_2_). The height is also equal to the water depth and, most notably, to the average depth of the ‘swimming pool’ (note that average depths and their variations with time are often the only pieces of information available for natural lakes).

In the ‘swimming pool’ geometry, the loss of volume is directly proportional to the decrease in average depth during evaporative water loss. Such an oversimplified scenario, however, would hardly occur in the natural environment.

Another possibility is a conical shape, as shown in Figure 1B. In this case, the surface area decreases with ongoing water evaporation, and it is possible to define a scale factor α so that *r*_2_ = α *r*_1_ and *h*_2_ = α *h*_1_. The volume of a cone is *V* = ⅓ π *r*^2^ *h*, where *d* = ⅓ *h* can be defined as the average water depth, and it is also *d*_2_ = α *d*_1_. On this basis, one gets *V*_2_ = ⅓ π *r*_2_^2^ *h*_2_ = α^3^ *V*_1_. The proportionality between depth decrease and volume loss is no longer linear but rather *V*_2_/*V*_1_ = (*d*_2_/*d*_1_)^3^.

A further case could be that of a hemisphere of radius *r* (Figure 1C), for which the surface area also decreases with evaporation. The hemisphere volume is *V* = π *h*^2^ (*r* − ⅓ *h*). Assume that the maximum water depth *h* scales as *h* = α *r*_._ The average depth is *d* = ½ *h* +⅙ *h*^3^ (2*hr* − *h*^2^)^−1^, and it is *V*_2_/*V*_1_ = (3α^2^ − α^3^)/2 with *h*_2_ = α *h*_1_. The volume trend is again described by a cubic function but is more complex than for the case of conical shape. For the sake of simplicity, a scenario as per Figure 1B was here assumed, where *V*_2_/*V*_1_ = (*d*_2_/*d*_1_)^3^.

Note that changes in average depth affect the degree by which a water body is illuminated by sunlight [21,22], thereby impacting photochemical reaction kinetics. Moreover, changes in volume during evaporation produce proportional increases in the concentration of non-volatile and water-soluble solutes. In our case, the following assumptions were made: *(i)* As a volatile solute, dissolved O_2_ was assumed to evaporate together with water, and its concentration was hypothesized not to vary in the process; *(ii)* no precipitation of CaCO_3_ was assumed to take place during water evaporation (calcium carbonate undersaturation or [Ca^2+^] < 8.7 × 10^−5^ M [23]); thus, we hypothesized that HCO_3_^−^ and CO_3_^2−^ simply underwent concentration while the water volume decreased; *(iii)* while (C)DOM underwent evaporative concentration, no changes in its features, such as the DOC-normalized absorption spectrum, were assumed to occur.

### 2.2. Changes in CEC Photodegradation during Evaporative Water Concentration

Evaporative water concentration increases the levels of water components having photochemical significance, such as NO_3_^−^, NO_2_^−^, HCO_3_^−^, CO_3_^2−^, and DOC. These act as sources and/or scavengers of PPRIs, and some of them are able to screen radiation [22], with the potential to modify the photodegradation kinetics of contaminants. The effects on photodegradation were assessed by modeling, using the software APEX version 1.1. APEX (Aquatic Photochemistry of Environmental Xenobiotics) predicts photodegradation kinetics of dissolved compounds based on their photochemical reactivity (absorption spectrum, direct photolysis quantum yield, second-order reaction rate constants with ^•^OH, CO_3_^•−^, ^1^O_2_, and ^3^CDOM*) and on features of the water environment (sunlight spectrum and irradiance, water depth, DOC, and concentrations of NO_3_^−^, NO_2_^−^, HCO_3_^−^, and CO_3_^2−^) [24,25]. In particular, the spectrum of sunlight used here and its irradiance were intended to reproduce a fair-weather, mid-latitude summer day in the northern hemisphere (clear-sky 15 July, 45 °N). The latest version of APEX was used in this work [26], which can take evaporative water concentration into account as *V* ∝ *d*^3^.

**Figure 2:**
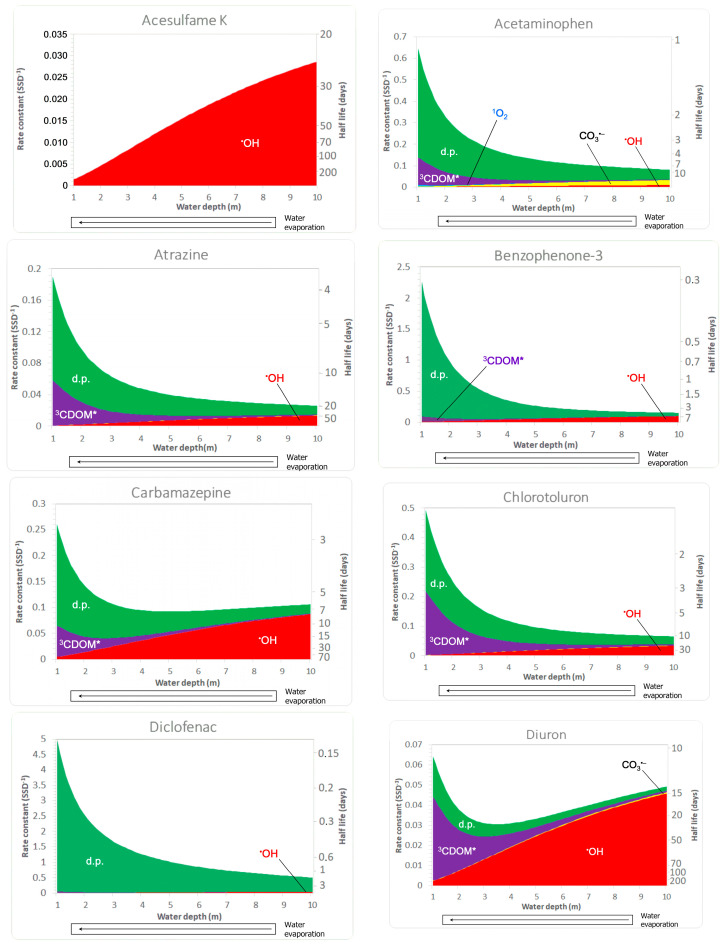

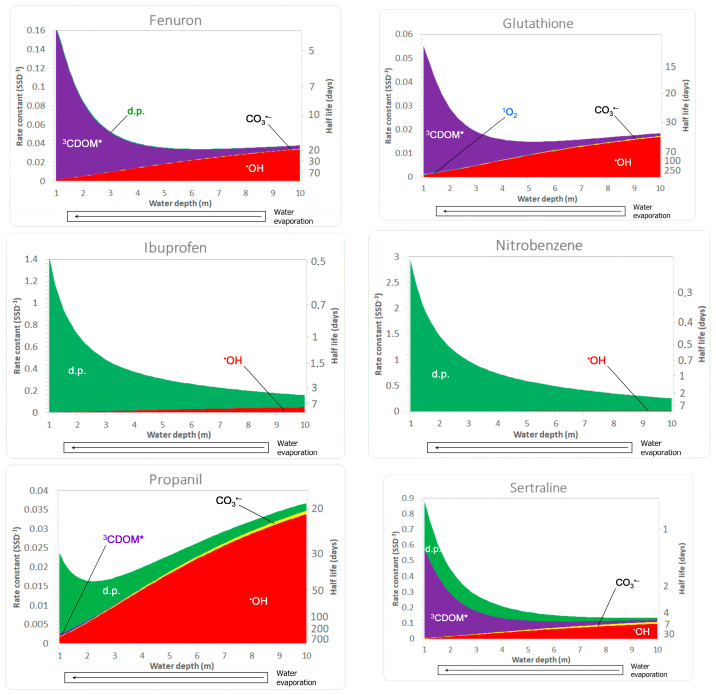
Predicted photodegradation kinetics of the considered CECs upon evaporative concentration of water. Left *Y*-axis: first-order photodegradation rate constants (*k*, SSD^−1^). Right *Y*-axis: corresponding half-life times (*t*_½_ = ln 2 *k*^−1^, days). Note that the acronym SSD (summer sunny day) is a time unit that indicates a fair-weather day corresponding to mid-latitude (45 °N) 15 July. The direction of water evaporation is in the sense of decreasing depth (see the arrows pointing from right to left). Initial water conditions (referred to the initial depth *d* = 10 m): 10^−6^ M NO_3_^−^, 10^−8^ M NO_2_^−^, 10^−5^ M HCO_3_^−^, 10^−7^ M CO_3_^2−^, 10^−9^ M Br^−^, and 0.1 mg_c_ L^−1^ DOC. Different colors highlight different photodegradation pathways (^•^OH, CO_3_^•−^, ^1^O_2_, ^3^CDOM*, d.p. = direct photolysis).

Simulation results are reported in Figure 2 for a range of compounds [26] that differ in photochemical reaction pathways and include pharmaceuticals (acetaminophen (paracetamol), carbamazepine, ibuprofen, and sertraline), pesticides (atrazine, chlortoluron, diuron, fenuron, and propanil), an artificial sweetener (acesulfame k), a solar filter (benzophenone-3), an industrial chemical intermediate (nitrobenzene), and a naturally occurring biological molecule (glutathione). The photochemical parameters (direct photolysis quantum yields, second-order reaction rate constants with PPRIs) of these compounds are listed in Table 1.

As shown in Figure 2, water evaporation would enhance direct photolysis and ^3^CDOM* reactions while inhibiting the processes induced by ^•^OH and CO_3_^•−^. Direct photolysis would benefit from the decrease in water depth because shallow water bodies are more thoroughly sunlit compared to deep ones. In the case of ^3^CDOM*, its only source (CDOM) would increase with evaporation, but the main sink (O_2_) would evaporate together with water, and its concentration would thus not increase. Increasing CDOM and constant O_2_ with evaporation account for the increasing importance of the ^3^CDOM* reactions predicted by the model [11], although DOM would become a significant ^3^CDOM* sink for DOC > 20 mg_C_ L^−1^. Although ^1^O_2_ would not play a key role in the degradation of the considered compounds, its reactions would undergo a similar enhancement as ^3^CDOM* because the ^1^O_2_ source (CDOM) would increase with evaporation while the ~55 M H_2_O concentration in aqueous solution would not be changed significantly by the assumed degree of evaporative concentration of the solutes [27].

In the case of ^•^OH, the concentration values of its photochemical sources (NO_3_^−^, NO_2_^−^, CDOM) would increase with evaporation, but the same would happen with the sinks (DOM, HCO_3_^−^, CO_3_^2−^, Br^−^, and NO_2_^−^). However, in the case of the ^•^OH sources, increasing their concentrations enhances photoreactions only up to the point when practically all sunlight is absorbed [11]. Beyond that, the enhancement effect levels off. In contrast, scavenging by the sinks is always directly proportional to their concentration [28]. Therefore, a parallel increase in both ^•^OH sources and sinks would be detrimental to ^•^OH-induced reactions. A similar issue holds for CO_3_^•−^; also note that CaCO_3_ precipitation, assumed here not to take place, would further inhibit the CO_3_^•−^ reactions by decreasing CO_3_^2−^, which yields CO_3_^•−^ when oxidized by ^•^OH or ^3^CDOM*.

Given the enhancement of ^3^CDOM*, ^1^O_2_, and direct photolysis and the inhibition of ^•^OH and CO_3_^•−^, the effect of water evaporation on the photodegradation of contaminants would depend on the prevailing reaction pathways that each compound undergoes in natural surface waters. For this reason, compounds that are mainly photodegraded by direct photolysis (benzophenone-3, diclofenac, ibuprofen, and nitrobenzene) or direct photolysis plus ^3^CDOM* (acetaminophen, atrazine, carbamazepine, chlortoluron, and sertraline) would undergo significant enhancement of photodegradation upon water evaporation. Moreover, similar enhancement is expected for other compounds that are also prevalently degraded by direct photolysis, such as the UV filter 2-ethylhexyl-4-(dimethylamino)benzoate and the ionic liquid 1-ethyl-3-methylimidazoliumhydrogensulfate [26]. In contrast, because ^•^OH-induced processes are inhibited, the photodegradation of compounds that react prevalently or exclusively with ^•^OH would be inhibited as well. This is particularly the case for the artificial sweetener acesulfame K (see Figure 2), which is one of the most refractory contaminants in surface waters [1,2].

In the cases of diuron and propanil, APEX predicts a pronounced minimum of their degradation rate constants at about 2–3 m depth (Figure 2). In such circumstances, initial inhibition of ^•^OH degradation by evaporation would be offset by the enhancement of direct photolysis and ^3^CDOM* reactions. For additional compounds (carbamazepine, fenuron, and glutathione), the contrasting inhibition/enhancement effects would grossly offset one another in the 5–10 m depth range, in which circumstances photodegradation kinetics would be approximately constant before increasing at *d* < 5 m.

Overall, a summary of the effects of water evaporation on the photodegradation of the studied compounds is provided in Table 2.

An important issue is that evaporative concentration of water would proportionally increase the aqueous levels of the investigated contaminants, which are all non-volatile or poorly volatile. The environmental impact of these compounds would thus increase, but in several cases, this would be at least partially offset by faster photodegradation.

There is a caveat, however, due to the possible formation of toxic intermediates in direct photolysis processes. Examples are the formation of 4-isobutylacetophenone from ibuprofen [11] and the formation of nitrophenols from nitrobenzene [29]. By contrast, water evaporation would decrease the potential for the production of toxic species when phenylurea herbicides (especially diuron and fenuron) react with ^•^OH [30,31].

An important increase in environmental occurrence is predicted for those compounds for which photodegradation is slowed down by evaporation, because increasing levels would be exacerbated by slower removal. This is especially the case for acesulfame K, plus propanil and diuron to somewhat lesser extents.

### 2.3. Possible Occurrence of Evaporative Concentration as a Consequence of Climate Change

The processes described above would take place in the case of significant water loss by evaporation. Current examples are some ephemeral lakes located in arid or semi-arid regions, which get replenished during the wet season and dry out in the dry season [32]. In particular, for evaporative water concentration to be effective, one needs very poor (or lack of) precipitation and elevated temperatures. Both events can be direct consequences of climate change, which could exacerbate existing conditions of water scarcity in several regions of the world [33,34].

We used a climate prediction tool developed by Columbia University (EdGCM, [35]) to assess possible evolutions of temperature and precipitation by the end of the century. The main input datum of the model is the time evolution of atmospheric CO_2_, which was here assumed to vary in the future as an extrapolation of the observed trend starting from 2000. In other words, we assumed a “business-as-usual” scenario.

It should be remarked that the EdGCM model predictions deal with average yearly values. This is particularly evident in the case of precipitation, for which a prediction of limited or no changes in yearly averages might hide a considerable modification of the precipitation regime during the year. Therefore, if no changes are foreseen in average precipitation for a region by the end of the century, it might either mean that rain in that region will have approximately the same features as now, or that the same average precipitation will be reached by means of long drought periods interspersed with a few very strong rain events. The latter scenario is already being observed in several regions of the world [36,37].

**Figure 3:**
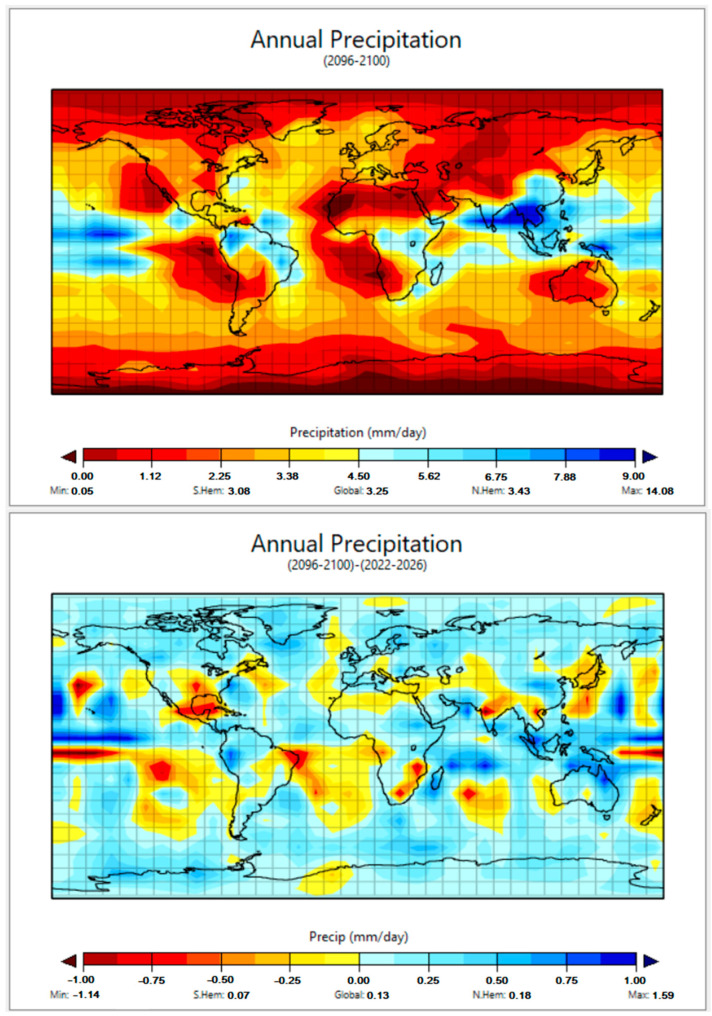
(**Top panel**): predicted global precipitation at the end of the century. (**Bottom panel**): predicted differences (increase or decrease) between global precipitation at the end of the century and the current situation.

In this framework, Figure 3 reports the predicted worldwide average precipitation at the end of the century (upper panel), as well as the differences from the present situation (lower panel). A first important issue is that worldwide average precipitation is expected to increase as a consequence of global warming. This is reasonable, considering that higher temperatures will favor oceanic water evaporation and that the global precipitation rate equals the rate of global evaporation [36]. Therefore, as shown in the lower panel of Figure 3, the zones in the world where average annual precipitation is expected to increase by the end of the century are more widespread than the areas for which the same parameter is expected to decrease.

However, the maps in Figure 3 also suggest that most of the increasing precipitation will occur over the global ocean or in near-equatorial areas, where current precipitation is already high. On the other hand, several areas that are already arid today might experience a decrease in average precipitation over time. This is, for instance, the case for northern Africa, parts of North America, and several regions in continental Asia. Interestingly, the EdGCM prediction is consistent with those of more sophisticated climate models [36,37]. A caveat is that water scarcity conditions might also be triggered by an irregular precipitation regime where, even at equal or quasi-equal average precipitation, extended drought periods alternate with strong precipitation events [37]. This scenario cannot be foreseen by the model we used, which only reports average values.

A decrease in average precipitation might favor the occurrence of evaporative water concentration, but it is not the only needed condition. An example is Indochina’s peninsula, where average precipitation is predicted to decrease, somewhere even considerably, but that is (and will still be) one of the rainiest regions on Earth (Figure 3).

The evaporation rate is also predicted by the EdGCM model, but this variable is little informative because it practically mirrors precipitation. Understandably, the highest evaporation is predicted to occur where it rains the most, but heavy rains are clearly not conducive to the evaporative concentration of water. In contrast, fair water evaporation in arid regions, where precipitation is low, can be enough to evaporatively concentrate a water body [38,39]. A substantial increase in temperature might thus be an important factor in predicting in which locations the phenomenon is most likely to occur.

**Figure 4:**
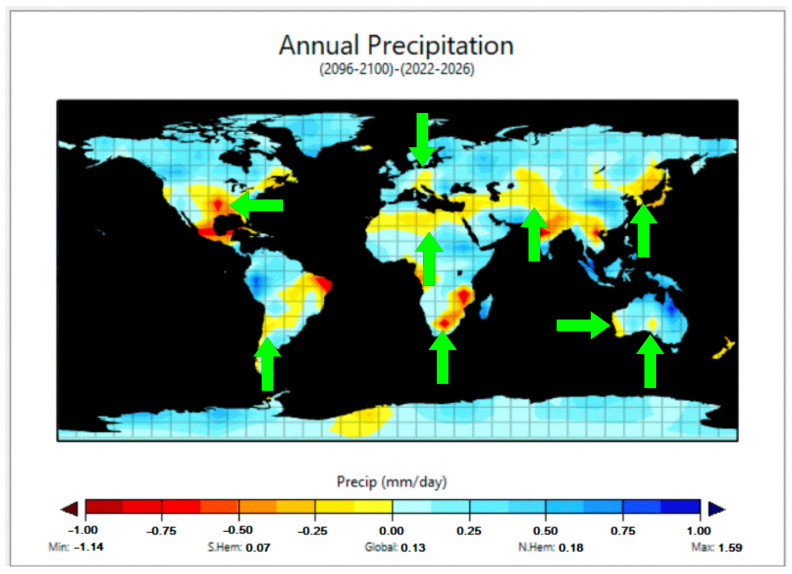

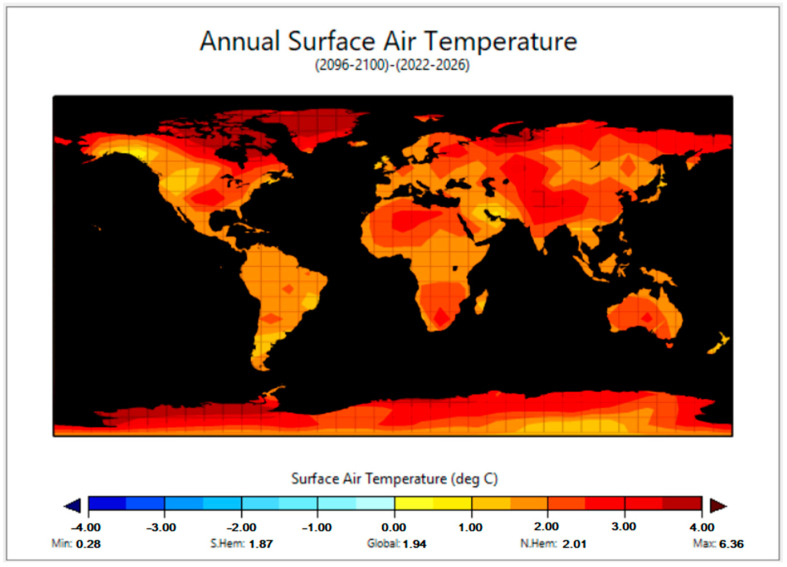
(**Top panel**): predicted differences between global precipitation at the end of the century and in the present time. (**Bottom panel**): corresponding differences as far as temperature is concerned. Only continental areas are considered in these maps. Arrows in the top panel highlight the areas with the highest potential for evaporative water concentration.

On this basis, Figure 4 reports predictions for both precipitation (upper panel) and temperature variations (lower panel). The EdCGM model results are shown, in both cases, as differences between the predicted scenarios that would occur by the end of the century and the present situation. To make figure reading easier, only data for continental areas are reported in Figure 4. In this framework, the regions where evaporative water concentration will be more likely are characterized by *(i)* an important degree of precipitation decrease (Figure 4, upper panel), *(ii)* low average precipitation levels (Figure 3, upper panel), and, at the same time, *(iii)* increasing temperature (Figure 4, lower panel). Such regions are highlighted by arrows in the upper panel of Figure 4. Interestingly, all the continents would be affected by the phenomenon in at least some areas. Therefore, the photochemical consequences of water evaporation should deserve particular attention in the future, because of important effects on pollutant attenuation combined with a predicted occurrence in several areas of the world.

## 3. Methods

### 3.1. APEX Software

The modeling of photochemical reaction kinetics and pathways was carried out with the APEX software (Aquatic Photochemistry of Environmental Xenobiotics) [26]. This software tool predicts the kinetics of each photochemical reaction pathway, as well as overall phototransformation, based on CEC reactivity parameters (absorption spectrum, direct photolysis quantum yields, second-order reaction rate constants with ^•^OH, CO_3_^•−^, ^1^O_2_, and ^3^CDOM*) and on environmental features (spectral irradiance of sunlight, water depth and absorption spectrum, DOC, and concentration values of NO_3_^−^, NO_2_^−^, HCO_3_^−^, CO_3_^2−^, and Br^−^). APEX uses a standard sunlight spectrum that corresponds to fair-weather, mid-morning, mid-July conditions at mid latitude and, for this reason, the standard time unit for both first-order degradation rate constants and half-life times is fair-weather 15 July at 45° N latitude. APEX also has a tool to predict seasonal variations in photoreaction kinetics, which was not used in this work. We used instead the procedure that takes into account the evaporative concentration of water and calculates the increase in concentration of NO_3_^−^, NO_2_^−^, HCO_3_^−^, CO_3_^2−^, Br^−^, and the DOC as the water depth decreases because of evaporation. As initial conditions, we used *d* = 10 m as well as 10^−6^ M NO_3_^−^, 10^−8^ M NO_2_^−^, 10^−5^ M HCO_3_^−^, 10^−7^ M CO_3_^2−^, 10^−9^ M Br^−^, and 0.1 mg_c_ L^−1^ DOC. By so doing, we could model the impact of water evaporation on the phototransformation pathways (direct photolysis, ^•^OH, CO_3_^•−^, ^1^O_2_, and ^3^CDOM*) of several CECs having known photoreaction kinetics. All the studied CECs are included in the APEX library that provides absorption spectra, photolysis quantum yields, and second-order reaction rate constants [26].

### 3.2. The EdGCM Global Climate Model

We used the EdGCM (Educational Global Climate Modeling) suite, version 3.1.1, to assess possible future climatic changes. The EdGCM software is a user-friendly modeling suite developed by Columbia University and based on the NASA/GISS global climate model [35]. The EdGCM suite needs as input the actual time trends of greenhouse gases rather than emission scenarios. Users should thus provide the time trends of CO_2_ and other climate-impacting gases, on the basis of which the software calculates the future evolution of parameters such as temperature, cloud cover, runoff, wind speed, snow and ice coverage, precipitation, and evaporation. Actual and future values of these parameters, as well as differences between years or between different year intervals, can be visualized as global maps.

**Figure 5:**
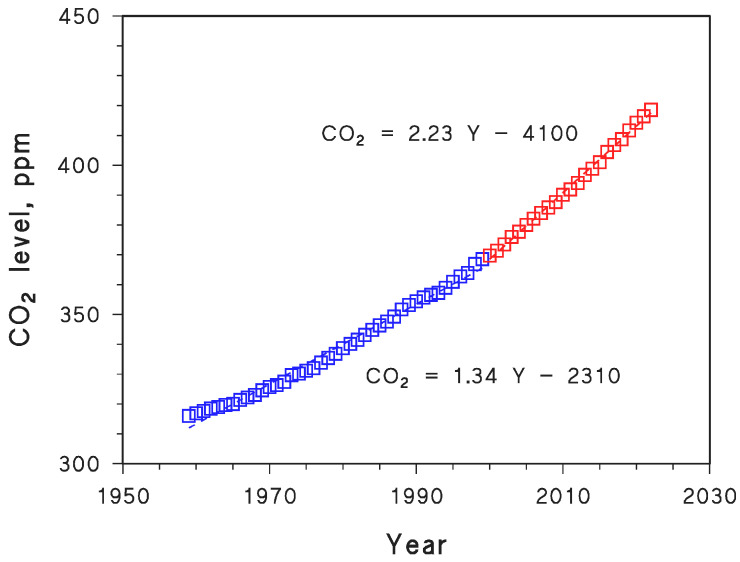
Measured global trend of atmospheric CO_2_ [45]. Different linear fits (see equations next to data points) were taken for the years 1959–1999 and after 2000. Note the increase in the slope of line after the year 2000.

When running, the EdGCM software first makes a control by comparing the predicted oceanic and atmospheric conditions with the actual values of the 1950s–1980s. For this reason, entries of atmospheric CO_2_ levels started from 1959. Then, considering the acceleration of CO_2_ emissions over the last couple of decades [40], such levels were divided into two time periods: the first ranged from 1959 to 1999, and the second from 2000 to the present (Figure 5). The equation derived during the second time period was extrapolated into the future by assuming a business-as-usual scenario. That might sound like a pessimistic guess, but it should be considered that the CO_2_ trend after 2000 resulted from actual emissions that were higher than the most pessimistic scenarios elaborated during the 1990s. Moreover, such a choice could also partially account for hard-to-predict phenomena that include the emission of other greenhouse gases due to positive feedback effects. One such example could be the emission of methane from thawing permafrost [41,42,43]. In our case, as the trends of methane, N_2_O, and hydrofluorocarbons are quite difficult to foresee, we assumed the atmospheric levels of these substances not to change in the future compared to the present levels.

Counterintuitively, future trends of water evaporation are not useful for our purposes because the global maps of evaporation closely mirror those of precipitation. In other words, evaporation is and will be the highest in rainy regions because a large amount of precipitation is needed to provide water to sustain high evaporation levels [44]. Conversely, evaporation rates in arid regions would not be high because there is not enough available water there, although these are the environments where evaporative concentration of surface waters is most likely to occur. The evaporative concentration phenomenon is, in fact, typical of water-scarcity conditions. For this reason, we looked for regions where scarce precipitation is predicted together with an above-average temperature increase. In these circumstances, we can imagine that little rain is available to dilute surface waters, while evaporation of the limited amount of occurring water is favored by elevated temperatures.

We have run the EdGCM model until the end of the 21st century. To limit variability in model output, we considered averages of 5-year periods. Therefore, differences between future and actual conditions were calculated between 2096–2100 and 2022–2026.

## 4. Conclusions

By means of photochemical modeling, we show here that evaporative water concentration would enhance the photochemical degradation of compounds that mainly undergo direct photolysis and reaction with ^3^CDOM*, while it should inhibit the degradation of compounds that mainly react with ^•^OH and CO_3_^•−^. Examples of CECs, the photodegradation of which would be enhanced are acetaminophen, atrazine, benzophenone-3, chlortoluron, fenuron, ibuprofen, nitrobenzene, and sertraline. In these cases, enhanced photodegradation could partly offset the increased impact arising from higher CEC occurrence due to evaporative concentration. However, faster photodegradation by direct photolysis could also enhance the formation of more harmful compounds from contaminants such as ibuprofen, diclofenac, and nitrobenzene. The occurrence of the intermediates would be enhanced considerably by the fact that the parent compounds reach high concentration values [11], which implies that in such scenarios, particular attention should be focused on harmful phototransformation intermediates.

In possibly fewer cases, photodegradation would be slowed down by evaporative water concentration. It is particularly the case for acesulfame K, and partially for propanil as well. In such cases, higher environmental occurrence would combine with enhanced persistence to increase the overall environmental effects. The photochemical implications of water evaporation would be less straightforward (either degradation enhancement or inhibition, depending on conditions) for compounds such as diuron.

These phenomena are likely to be exacerbated by ongoing climate change. In particular, it appears that at least some locations on all continents could be affected by evaporative water concentration, due to the combination of decreasing precipitation and increasing temperatures.

## Figures and Tables

**Table 1:** Photochemical reaction parameters of the modeled CECs (direct photolysis quantum yields, Φ_d.p._, and second-order reaction rate constants, *k*_PPRI_) [26]. E = Einstein (mole of photons).

Compound	Φ_d.p._ (mol E^−1^)	Reaction Rate Constants (M^−1^ s^−1^)			
		*k*_°OH_	*k*_CO₃°‾_	*k* ^1^_O_2__	*k* ^3^_CDOM*_
Acesulfame K	Negligible	5.9 × 10^9^	Negligible	2.8 × 10^4^	Negligible
Acetaminophen ^1^	4.6 × 10^−2^	1.9 × 10^9^	3.8 × 10^8^	3.7 × 10^7^	1.6 × 10^9^
Atrazine	1.6 × 10^−2^	2.7 × 10^9^	4 × 10^6^	Negligible	7.15 × 10^8^
Benzophenone-3	3.1 × 10^−5^	2.0 × 10^10^	Negligible	2.0 × 10^5^	1.1 × 10^9^
Carbamazepine	7.8 × 10^−4^	1.8 × 10^10^	Negligible	1.9 × 10^5^	7.5 × 10^8^
Chlortoluron	3 × 10^−2^	6.9 × 10^9^	1.7 × 10^7^	Negligible	2.7 × 10^9^
Diclofenac	9.4 × 10^−2^	9.3 × 10^9^	Negligible	1.3 × 10^7^	6.4 × 10^8^
Diuron	1.25 × 10^−2^	9.45 × 10^9^	8.3 × 10^6^	Negligible	5.2 × 10^8^
Fenuron	6 × 10^−3^	7 × 10^9^	6.0 × 10^6^	Negligible	2.0 × 10^9^
Glutathione	Negligible	3.5 × 10^9^	5.3 × 10^6^	2.4 × 10^6^	6.7 × 10^8^
Ibuprofen	0.33	1.0 × 10^10^	Negligible	6.0 × 10^4^	4.5 × 10^7^
Nitrobenzene	5.7 × 10^−3^	3.9 × 10^9^	Negligible	Negligible	1.1 × 10^8^
Propanil	0.16	7.0 × 10^9^	1.4 × 10^7^	7.1 × 10^4^	1 × 10^7^
Sertraline	0.95	2 × 10^10^	2 × 10^8^	1.3 × 10^6^	7 × 10^9^

^1^ Also known as Paracetamol.

**Table 2:** Effects of water evaporation on the photodegradation of the compounds under study.

Compound	Effect of Water Evaporation on Photodegradation Rates
Acesulfame K	Decrease
Acetaminophen	Increase
Atrazine	Increase
Benzophenone-3	Increase
Carbamazepine	Increase if water depth reduces to less than one third
Chlortoluron	Increase
Diclofenac	Increase
Diuron	Little change
Fenuron	Increase if water depth reduces to less than one half
Glutathione	Increase if water depth reduces to less than one half
Ibuprofen	Increase
Nitrobenzene	Increase
Propanil	Mainly decrease
Sertraline	Increase

## Data Availability

Data supporting this study can be provided by the authors on request.
